# Dectin-1 predicts adverse postoperative prognosis of patients with clear cell renal cell carcinoma

**DOI:** 10.1038/srep32657

**Published:** 2016-09-07

**Authors:** Yu Xia, Li Liu, Qi Bai, Jiajun Wang, Wei Xi, Yang Qu, Ying Xiong, Qilai Long, Jiejie Xu, Jianming Guo

**Affiliations:** 1 Department of Urology, Zhongshan Hospital, Fudan University, Shanghai 200032, China; 2 Department of Biochemistry and Molecular Biology, School of Basic Medical Sciences, Fudan University, Shanghai 200032, China

## Abstract

Dectin-1, a classical pattern-recognition receptor, was now identified as an important regulator in immune homeostasis and cancer immunity through its extensive ligands binding functions and subsequent cytokines production. The aim of this study was to assess the clinical significance of dectin-1 expression in 290 patients with clear cell renal cell carcinoma (ccRCC) through immunohistochemistry on tissue microarrays. We found that dectin-1 was predominantly expressed on ccRCC cells, in accordance with several other online databases. Moreover, Kaplan-Meier method was conducted and high expression of tumoral dectin-1 was associated with shorter patient recurrence free survival (RFS) and overall survival (OS) (P < 0.001 for both). In multivariate analyses, tumoral dectin-1 expression was also confirmed as an independent prognostic factor for patients’ survival together with other clinical parameters (P < 0.001 for RFS and OS). After incorporating these characteristics including tumoral dectin-1 expression, two nomograms were constructed to predict ccRCC patients’ RFS and OS (c-index 0.796 and 0.812, respectively) and performed better than existed integrated models (*P* < 0.001 for all models comparisons). In conclusion, high tumoral dectin-1 expression was an independent predictor of adverse clinical outcome in ccRCC patients. This molecule and established nomograms might help clinicians in future decision making and therapeutic developments.

Renal cell carcinoma (RCC) accounts for about 2–3% of all adult malignancies and causes approximately 102,000 deaths around the world every year. Nearly one third of the patients who underwent curative surgeries would develop recurrences or metastases afterwards^1^. Owing to complicated molecular heterogeneity in tumors, current TNM stage, Fuhrman grade and several integrated models were not enough for RCC patient outcome prediction^2^. Incorporating specific molecular biomarkers into existing models might help solve this problem and numerous studies focusing on the genetic and proteome signature of RCC are underway over the world^3^.

Dectin-1 (official name CLEC7A), belonging to the c-type lectin superfamily, was originally identified as a pattern-recognition receptor (PPR) expressed on dendritic cells (DCs)^4^, specifically against exogenous fungal pathogens with β-glucan structure^5^. It contains an immunoreceptor tyrosine-based activation motif (ITAM)-like motif, which is generally involved in immune cell activation and cytokine production^6^. However, recent published papers suggested its extensive ligand structures and comprehensive immunomodulation function in different cell types. Like other PRRs, dectin-1 has several endogenous ligands, such as some unidentified N-glycans^7^, intestinal mucin-2^8^ and costimulatory molecule on T-cells^9^. Dectin-1 could interact with these ligands and regulate downstream immune homeostasis, autoimmunity, allergy and cancer immunity^10^. Dectin-1 expressed on DCs and macrophages was shown to recognize the N-glycans on tumor cells and enhance tumour killing by natural killer (NK) cells through homophilic interactions, suggesting its potential role in cancer immunomodulation^7^.

In recent years, several immunotherapies focusing on immunoreceptors such as PD-1 or CTLA-1 have brought great success in RCC treatment, and it was widely accepted that a dynamic interplay existed between the host and tumor^11^. Cancer cells could shape its microenvironment into a pro-tumor type through the modulation on infiltrated immune cells^12^, and we wondered whether dectin-1 could participate in such process and become a potential prognostic marker for RCC patients. Thus, here through immunohistochemistry, we investigated the expression of intratumoral dectin-1 in a large cohort of clear cell RCC patients (ccRCC, the most common histologic type of RCC) and analyzed the impact of dectin-1 expression on their recurrence-free survival (RFS) and overall survival (OS).

## Results

### Dectin-1 staining intensity and its association with clinicopathological characteristics

**Figure 1:**
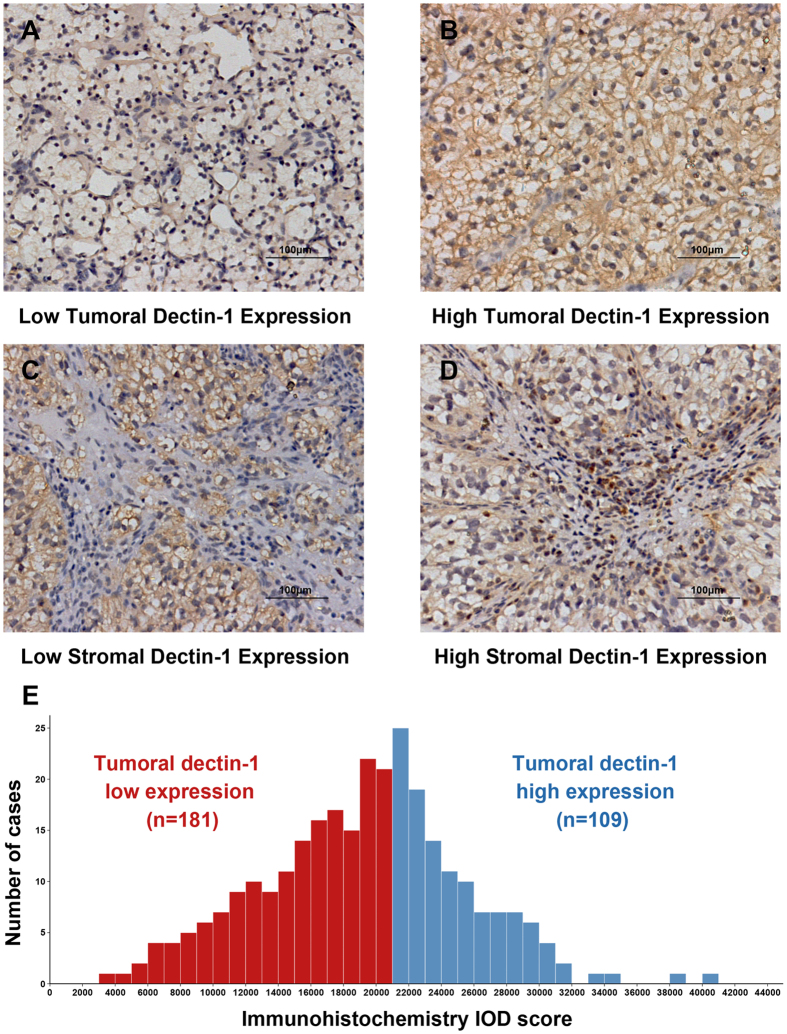
Representative photographs of dectin-1 immunostaining in ccRCC. (**A**) Tumoral dectin-1 low expression; (**B**) Tumoral dectin-1 high expression; (**C**) Stromal dectin-1 low expression; (**D**) Stromal dectin-1 high expression. Scale bar = 100 μm. Original magnification ×200. (**E**) Frequency distribution of tumoral dectin-1 immunohistochemistry integrated optical density (IOD) score in 290 ccRCC samples.

Dectin-1 expression was predominantly found on the membrane and cytoplasm of tumor cells and the intensity of the staining was variable (Fig. 1A,B). In the stromal areas, there were also some positive cells aggregation (Fig. 1D), but due to the complexity of stromal cell composition, we did not further explore its significance on patient survival. For tumoral dectin-1 expression, the staining intensity was divided into low and high based on the cut-off value (21000) derived from total IOD score (median 19761, IQR 15272-23018; mean and SD, 19249 ± 6177) and minimum p value method (Fig. 1E). Tumoral dectin-1 expression was strongly associated with higher pT stage (*P* = 0.031), while its correlation with other clinicopathological characteristics did not meet statistical significance (Table 1).

### Clinical outcomes and association of tumoral dectin-1 expression with survival

**Figure 2:**
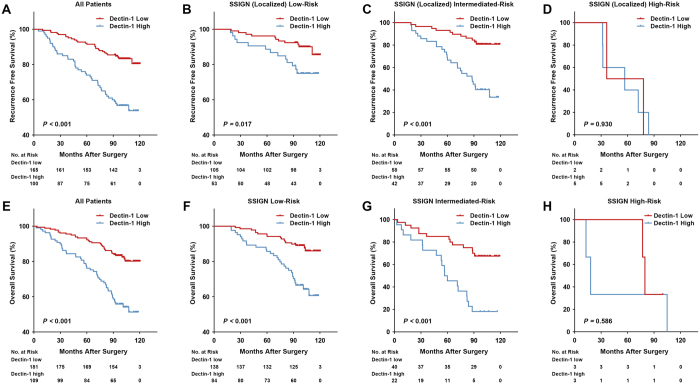
Tumoral dectin-1 expression stratified by SSIGN (localized)/SSIGN score and related Kaplan-Meier analyses of patient recurrence free survival (RFS) and overall survival (OS). (**A**) RFS of all ccRCC patients according to tumoral dectin-1 expression; (**B**–**D**) RFS of patients in different SSIGN (localized) risk groups according to tumoral dectin-1 expression; (**E**) OS of all available ccRCC patients according to tumoral dectin-1 expression; (**F**–**H**) OS of patients in different SSIGN risk groups according to tumoral dectin-1 expression. *P-value*, calculated by log rank test, <0.05 was regarded as statistically significant.

The median follow-up for all available patients was 99.03 months (range 2.63–120.47). 83 in 290 patients (28.6%) died during the follow up and 72 in 265 patients (27.2%) experienced disease relapse after excluding those with missing data or preoperational metastasis. Kaplan-Meier survival curves were carried out to analyze the RFS and OS according to dectin-1 expression. As is shown in Fig. 2A,E, the median survivals for both the low and high dectin-1 expression groups in the RFS and OS analyses were not reached during the follow-up. The mean survival estimates for RFS were 109.8 months (SE, 2.0, 95% CI, 105.9–113.7) for dectin-1 low expression and 88.7 months (SE, 3.8, 95% CI, 81.2–96.2) for high expression. In OS analysis, they were 109.6 months (SE, 1.9, 95% CI, 105.9–113.3) and 90.1 months (SE, 3.4, 95% CI, 83.4–96.9), respectively. Patients with high tumoral dectin-1 expression had a significantly poorer RFS (*P* < 0.001) and OS (*P* < 0.001). This result was further confirmed by univariate analysis, in which high tumoral dectin-1 expression was significantly associated with poor patient survivals (RFS, HR, 3.139, 95% CI, 1.953–5.045, *P* < 0.001; OS, HR, 3.053, 95% CI, 1.966–4.742, *P* < 0.001) (Table S1). Furthermore, in multivariate analysis, high tumoral dectin-1 expression was also an independent predictor for both RFS and OS (RFS, HR, 2.436, 95% CI, 1.494–3.970, *P* < 0.001; OS, HR, 3.123, 95% CI, 1.961–4.976, *P* < 0.001). After a 1000-resampled bootstrap correction, its significance remained (RFS, HR, 2.438, 95% CI, 1.276–4.367, *P* = 0.006; OS, HR, 3.212, 95% CI, 1.862–5.906, *P* = 0.001), together with pT stage, distant metastasis, Fuhrman grade, necrosis and Eastern Cooperative Oncology Group Performance Status (ECOG PS) (Table 2).

### Predictive impact of tumoral dectin-1 expression upon SSIGN/SSIGN(localized) and UISS model

The Mayo Clinic stage, size, grade and necrosis score (SSIGN) score was applied to classify patients into three risk levels: 0–3 (low), 4–7 (intermediate), ≥8 (high) for OS analysis, and the SSIGN localized (Leibovich) score: 0–2, 3–5, ≥6 for RFS analysis^13^,^14^. As is seen in Fig. 2, high tumoral dectin-1 expression displayed as a poor prognostic factor in the low- and intermediated-risk groups in both RFS and OS analyses (RFS, *P* = 0.017 (median survival estimates 114.3 months, SE, 1.9, 95% CI, 110.5–118.1 for low expression and 103.7 months, SE, 4.0, 95% CI, 95.8–111.6 for high expression), OS, *P* < 0.001 (median survival estimates 113.6 months, SE, 1.7, 95% CI, 110.3–116.9 for low expression and 98.7 months, SE, 3.3, 95% CI, 92.2–105.2 for high expression) in low-risk groups; RFS, *P* < 0.001 (median survival estimates 102.9 months, SE, 4.0, 95% CI, 95.1–110.7 for low expression and 75.0 months, SE, 6.1, 95% CI, 63.1–86.9 for high expression), OS, *P* < 0.001 (median survival estimates 97.2 months, SE, 5.7, 95% CI, 85.9–108.4 for low expression and 62.6 months, SE, 7.5, 95% CI, 47.8–77.3 for high expression) in intermediated-risk groups), while in the high-risk groups it did not meet statistical significance possibly owning to the small sample size. Moreover, tumoral dectin-1 expression information could add additional prognostic power for SSIGN (localized) score system in patients’ RFS prediction (c-index 0.762 *vs* 0.718, *P* = 0.017). For the University of California Integrated Staging System (UISS) score^15^, adding tumoral dectin-1 expression into the model could also strengthen its accuracy for both RFS and OS prediction (c-index 0.760 *vs* 0.713, *P* = 0.010 for RFS; c-index 0.763 *vs* 0.723, *P* = 0.050 for OS) (Table 3).

### Construction and validation of prognostic nomogram for OS and RFS

**Figure 3:**
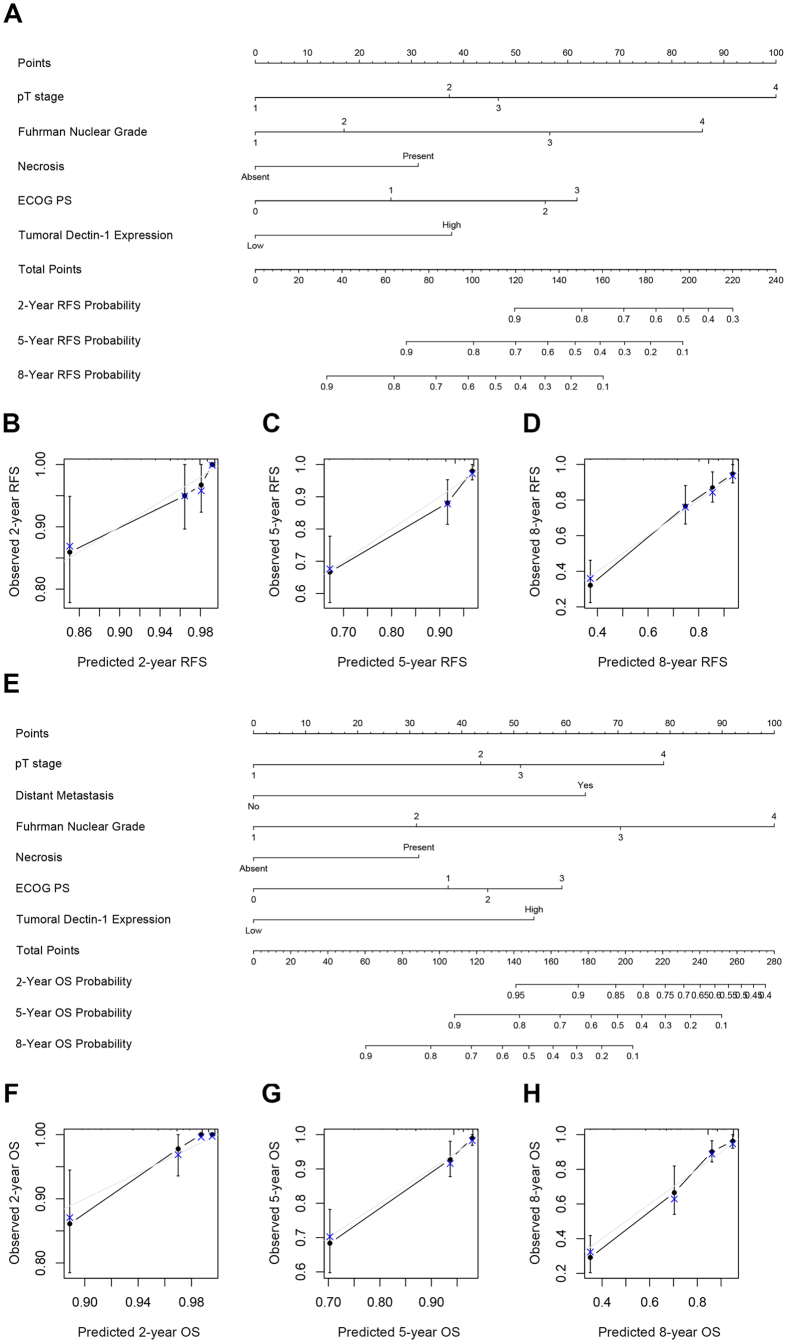
Nomogram for predicting 2-, 5- and 8-year recurrence free survival (RFS) and overall survival (OS) in patients with ccRCC. (**A**) Nomogram for predicting RFS integrating pT stage, Fuhrman nuclear grade, necrosis, ECOG PS and tumoral dectin-1 expression; (**B**–**D**) Calibration plot for predicted and observed 2-, 5- and 8-year RFS rate; (**E**) Nomogram for predicting OS integrating pT stage, distant metastasis, Fuhrman nuclear grade, necrosis, ECOG PS and tumoral dectin-1 expression; (**F**–**H**) Calibration plot for predicted and observed 2-, 5- and 8-year OS rate. *The grey line:* ideal model, *vertical bars:* 95% confident interval.

Two nomograms for predicting 2-, 5- and 8-year ccRCC patients’ RFS and OS were established based on the validated multivariate analyses (Fig. 3A,E), involving pT stage, distant metastasis, Fuhrman grade, necrosis status, ECOG PS and tumoral dectin-1 expression. A value was assigned to each level of these variables, and the total values could be used to estimate patient survival probability at different time. Bootstrap validations were performed to exam the robustness of these models and the calibration plots displayed good consistency between the predicted and actual observation of patient survival (Fig. 3B–D,F–H). The Harrell’s c-index was 0.796 (95% CI, 0.747–0.846) and 0.812 (95% CI, 0.769–0.856) for RFS and OS prediction respectively. After comparing the c-indexes of established nomograms with those of the SSIGN(localized)/SSIGN and UISS models, we found that the two nomograms presented significant advantages in both RFS and OS prediction (*P* < 0.001). These superior performances were also kept among SSIGN(localized)/SSIGN and UISS defined low/intermediate risk patients (*P* < 0.001) (Table 3).

## Discussion

Up to now, more than 5600 separate reports focusing on RCC prognostic markers have been published. However, in routine clinical usage, no such molecule has been verified applicable and remarkable for the survival assessment of RCC patients^3^. It has been widely accepted that cancer immunoediting and subsequent immune escape played an important role in tumor progression^12^, and several recent articles also reported that the molecular signatures involving inflammation and immune response in RCC were related to patient survival and drug resistance^16^,^17^,^18^. Thus, exploring the prognostic roles of these immune related molecules might help RCC patient survival prediction and new agent development.

In this study, we focused on a classical PPR, dectin-1, and its expression in RCC samples through immunohistochemistry. We found that dectin-1 was predominantly expressed on tumor cells. Moreover, high tumoral dectin-1 expression positively correlated with higher pT stage and could be used as an independent prognosticator in ccRCC patients’ RFS and OS prediction after adjusted with other parameters. Adding tumoral dectin-1 information into existed models such as TNM, SSIGN and UISS would noticeably enhance their prognostic power. Finally, after incorporating tumoral dectin-1 with other clinical parameters, two nomograms were generated to predict patients’ RFS and OS, and performed better than existed prognostic models by c-indexes comparison.

Dectin-1 was originally thought to be a DC specific receptor, from which its name ‘dendritic-cell-associated C-type lectin-1’ was derived^4^, and could protect host from fungus infection through β-glucan recognition^5^. This receptor is now known to be expressed by many other immune cells, such as macrophages, monocytes, neutrophils, eosinophils, T cells and B cells and has many physiological roles like immune homeostasis and cancer immune regulation^10^. In our research, however, dectin-1 expression was predominantly found on tumor cells, and this result was in accord with many other online databases. Referring to oncomine, we found that 4 in 5 ccRCC studies confirmed a higher expression of dectin-1 mRNA compared to normal tissues. The DNA information from 2013 TCGA cohort data also suggested amplification of dectin-1 copies in ccRCC samples. It is not rare that tumor cells could aberrantly express some immune receptors and facilitate immune regulation function, like the PD-L1 expressed on tumor cells and its inhibition on PD-1 positive cells^19^. And dectin-1 did have several endogenous ligands, such as some unidentified molecules on T-cells, glycosidoprotein mucin-2 and N-glycans. It has been recognized that dectin-1 could bind to these ligands and alter T cells function or NK cells’ anti-tumor response^7^,^8^,^9^. Thus, we speculate that the aberrant dectin-1 expression on ccRCC might also interact with these identified or unidentified ligands on immune cells and interfere with their tumor surveillance function.

Dectin-1 is an activation receptor, transducing intracellular signals via an ITAM-like motif within its cytoplasmic tail^6^. This signal could basically stimulate immune cell maturation and proliferation, and induce numerous cytokines and chemokines production including TNF, CXCL2, IL-23, IL-6, IL-10 and IL-2, which are cell type dependent^20^. As dectin-1 was found predominantly on RCC cells in our study, tumor cell might also be activated through this signal and facilitate specific immune regulatory cytokine production such as IL-10 and IL-23. Moreover, some studies found that macrophage treated with IL-4 or IL-13 (alternatively activated macrophage) could express high levels of dectin-1^21^, suggesting that these immune regulatory cytokines might also influence tumor cell dectin-1 expression, like a tumor-host cytokine interaction.

In summary, our group has identified that dectin-1 was preponderantly expressed by ccRCC tumor cells and exerted profound impact on patient survival. This finding may also indicate this molecule as a potential target for future immune therapies development. However, there were also several limitations in this study, one of which was its retrospective and single-centered design in nature. Though bootstrap has been performed for minimizing overfitting bias, the cut-off point chosen for tumoral dectin-1 expression and its prognostic significance in ccRCC patients should still be concerned and validated in further external cohorts. Moreover, the proportion of advance ccRCC patients were much smaller in our study which hindered the robustness of this prognostic marker in such patient groups. A prospective, multicenter study is needed, and researches are also required to investigate the detailed roles of dectin-1 in either ccRCC tumor cells or surrounding positive immune cells.

## Methods

### Patient selection

This study included 290 ccRCC patients who underwent nephrectomy in the Department of Urology, Zhongshan Hospital, Fudan University between Jan 2005 and Jun 2007. All methods mentioned below were approved by the ethics committee of Zhongshan Hospital with the approval number B2015-030 and were carried out in accordance with the approved guidelines. Written informed consent on the use of clinical specimens from each patient was achieved. The primary inclusion criteria were patients (1) having pathologically proven ccRCC (2) having received partial or radical nephrectomy and (3) having available Formalin Fixed Paraffin Embedded (FFPE) specimen of tumor mass (≥1cm^3^). Those who had other former malignant tumor, perioperative mortalities, histories of adjuvant or neoadjuvant targeted therapies and patients with mixed type renal cancer, bilateral renal cancer and FFPE samples necrosis area >80% were excluded.

### Data collection

Patients’ RFS was counted from the time of nephrectomy to the time of recurrence (defined as local or distant metastases confirmed by imaging, biopsy or physical examination), and OS was calculated from the time of nephrectomy to the time of death. The follow-up interval was three months during the first 5 years and annually thereafter. Information was censored if patient died without recurrence or lived till the last follow up time (Jan 30, 2015). 7 patients were excluded from RFS analysis for missing data of recurrence state and 18 patients for preoperational metastases. All baseline demographic, clinical, and laboratory data were reconfirmed. MRI and CT scans were reassessed by radiology units, and diagnostic H&E slides were reviewed by two urologic pathologists independently (Yuan J. and Jun H.). According to the 2014 EAU guidelines, ccRCC histologic subtype was confirmed^22^. Tumor stage was reclassified based on the 2010 AJCC TNM classification^23^. Fuhrman grade and coagulative necrosis were reported according to 2012 ISUP consensus^3^. The SSIGN, SSIGN localized (Leibovich) and UISS score were applied to stratify patient risks as previously reported^13^,^14^,^15^.

### Immunohistochemistry and evaluation

Immunohistochemical staining was performed on tissue microarray (two cores for one tumor block) with appropriate antibodies (Anti-Dectin-1 antibody, ab140039, Abcam, diluted 1/100) and visualization reagent (Dako EnVision Detection System) as previously described^24^. The specificity of antibody was confirmed by western blot using RCC cell lines. IHC procedures without applying the primary antibody were conducted as negative control. Olympus CDD camera, Nikon eclipse Ti-s microscope (×200 magnification) and NIS-Elements F3.2 software were used to record the staining results and three independent shots with strongest staining were selected for each tumor core. The integrated optical densities (IOD) scores for each scan were calculated by Image-Pro Plus version 6.0 software (Media Cybernetics Inc., Bethesda, MD, USA) and the pooled IOD mean of the six spots in two tumor cores was regarded as the final staining intensity for each block. One urologic pathologist unaware of the patients’ clinical features and outcomes evaluated these slides. The IOD score cut-point for determining tumoral dectin-1 high/low expression was evaluated by X-tile software through minimum p value method^25^.

### Statistical analysis

χ2 test, Fisher’s exact method and Cochran-Mantel-Haenszel χ2 test were applied for assessing the relationship between tumoral dectin-1 expression and patients’ clinicopathological parameters. RFS and OS curves were illustrated using Kaplan–Meier method and log-rank test was applied for comparison between different groups. Cox univariate analysis was carried out and those parameters with statistical significance were brought into a multivariate Cox proportional hazards model and formed two nomograms for predicting patients’ RFS and OS. Concordance index (c-index) were generated to assess the predictive accuracy and sufficiency of different models, while Hanley-McNeil test was applied to compare the difference between c-indexes. 1000 bootstrap resamples were performed in multivariate analyses and c-index calculations for reducing overfitting bias. GraphPad Prism 6 (GraphPad Software Inc., La Jolla, CA, USA), SPSS 21.0 (SPSS Inc., IL, Chicago, USA), Stata (version 12.1; StataCorp LP, TX, USA) and R software version 3.1.2 with the “rms” package (R Foundation for Statistical Computing, Vienna, Austria) were used in these procedures. A two-sided *P-value* <0.05 was regarded as statistically significant.

## Additional Information

**How to cite this article**: Xia, Y. *et al*. Dectin-1 predicts adverse postoperative prognosis of patients with clear cell renal cell carcinoma. *Sci. Rep.* **6**, 32657; doi: 10.1038/srep32657 (2016).

## Supplementary Material

Supplementary Information

## Figures and Tables

**Table 1:** Clinical characteristics of patients according to tumoral dectin-1 expression.

Characteristics	Patients		Tumoral dectin-1 expression		
	n	%	low	high	P-value
All patients	290	100	181	109	
Age, years*	range	15–86			0.250^†^
≤55	143	49.3	94	49	
>55	147	50.7	87	60	
Gender					0.958^†^
Female	91	31.4	57	34	
Male	199	68.6	124	75	
Tumor size, cm*					0.948^†^
≤4	163	56.2	102	61	
>4	127	43.8	79	48	
Pathological T stage					0.031^‡^
pT1	183	63.1	122	61	
pT2	27	9.3	16	11	
pT3	76	26.2	42	34	
pT4	4	1.4	1	3	
Pathological N stage					0.495^†^
pN0	35	12.1	19	16	
pN1	2	0.7	2	0	
pNx	253	87.2			
Distant metastasis					0.994^†^
No	274	94.5	171	103	
Yes	16	5.5	10	6	
TNM stage					0.098^‡^
I	177	61.0	117	60	
II	23	7.9	14	9	
III	70	24.1	39	31	
IV	20	6.9	11	9	
Fuhrman grade					0.557^‡^
1	31	10.7	19	12	
2	214	73.8	136	78	
3	42	14.5	25	17	
4	3	1.0	1	2	
Necrosis					0.058^†^
Absent	251	86.6	162	89	
Present	39	13.4	19	20	
ECOG PS					0.312^‡^
0	211	72.8	137	74	
1	64	22.1	34	30	
2	11	3.8	8	3	
3	4	1.4	2	2	

*Split at median; ^†^χ^2^ test or Fisher’s exact test, ^‡^Cochran-Mantel-Haenszel χ^2^ test, P**-**value <0.05 was regarded as statistically significant; ECOG PS = Eastern Cooperative Oncology Group performance status.

**Table 2:** Proportional hazard model for recurrence free survival and overall survival prediction.

Variables	RFS (n = 265)				OS (n = 290)			
	Base model		Bootstrap validate model*		Base model		Bootstrap validate model*	
	HR (95%CI)	P-value^†^	HR (95%CI)	P-value^†^	HR (95%CI)	P-value^†^	HR (95%CI)	P-value^†^
Pathological T stage		<0.001		0.001		<0.001		0.001
pT1	Reference	—	Reference	—	Reference	—	Reference	—
pT2 *vs* pT1	2.579 (1.170–5.681)	0.019	2.790 (1.080–6.760)	0.035	2.691 (1.331–5.443)	0.006	2.759 (1.185–6.341)	0.013
pT3 *vs* pT1	3.090 (1.786–5.346)	<0.001	3.114 (1.329–5.590)	0.004	2.986 (1.791–4.977)	<0.001	3.037 (1.675–5.635)	0.001
pT4 *vs* pT1	11.864 (3.743–37.605)	<0.001	13.183 (1.000–64.264)	0.001	5.740 (1.630–20.209)	0.007	5.737 (0.000–72.675)	0.054
Distant metastasis								
Yes *vs* No	—	—	—	—	4.089 (2.121–7.885)	<0.001	4.787 (1.822–15.379)	0.008
Fuhrman grade		<0.001		<0.001		0.001		0.001
1–2	Reference	—	Reference	—	Reference	—	Reference	—
3 *vs* 1–2	2.744 (1.502–5.012)	0.001	2.751 (1.023–5.414)	0.002	2.471 (1.414–4.317)	0.001	2.588 (1.439–4.563)	0.002
4 *vs* 1–2	5.675 (1.648–19.538)	0.006	5.906 (1.000–17.868)	0.001	4.782 (1.409–16.227)	0.012	5.328 (2.125–18.634)	0.004
Necrosis								
Present *vs* Absent	2.182 (1.211–3.930)	0.009	2.232 (1.012–4.477)	0.011	2.033 (1.146–3.607)	0.015	2.136 (1.136–4.242)	0.025
ECOG PS		0.003		0.031		0.003		0.010
0	Reference	—			Reference	—	Reference	—
1 *vs* 0	1.892 (1.093–3.273)	0.023	1.908 (1.005–3.636)	0.039	2.236 (1.383–3.615)	0.001	2.293 (1.302–4.047)	0.004
2 *vs* 0	3.954 (1.374–11.379)	0.011	3.304 (1.104–12.858)	0.025	2.658 (0.990–7.142)	0.052	2.477 (0.482–9.281)	0.104
3 *vs* 0	4.142 (1.283–13.367)	0.017	5.496 (0.947–32.852)	0.097	3.193 (0.890–11.455)	0.075	2.787 (0.320–15.721)	0.101
Tumoral dectin-1								
Low *vs* High	2.436 (1.494–3.970)	<0.001	2.438 (1.276–4.367)	0.006	3.123 (1.961–4.976)	<0.001	3.212 (1.862–5.906)	0.001

ECOG PS = Eastern Cooperative Oncology Group performance status; HR = hazard ratio; CI = confidence interval; OS = overall survival; RFS = recurrence free survival; ^†^Data obtained from the Cox proportional hazards model, P**-**value <0.05 was regarded as statistically significant; *Bootstrapping with 1000 resamples were used.

**Table 3:** Comparison of the predictive accuracy of the prognostic models.

Models	Recurrence free survival			Overall survival		
	C-index (95%CI)	Coefficient (95%CI)	P-value	C-index (95%CI)	Coefficient (95%CI)	P-value
Tumoral Dectin-1	0.643 (0.587–0.700)	—		0.638 (0.585 –0.691)	—	
TNM	0.658 (0.600–0.716)	—		0.702 (0.648–0.757)	—	
TNM + Tumoral Dectin-1	0.708 (0.650–0.767)	0.050 (0.012–0.089)	0.001^†^	0.747 (0.694–0.800)	0.045 (0.013–0.078)	0.006^†^
SSIGN	0.718 (0.665–0.772)	—		0.740 (0.692–0.787)	—	
SSIGN + Tumoral Dectin-1	0.762 (0.708–0.815)	0.044 (0.008–0.080)	0.017^†^	0.772 (0.723–0.821)	0.032 (−0.006–0.071)	0.093^†^
UISS	0.713 (0.662–0.764)	—		0.723 (0.670–0.776)	—	
UISS + Tumoral Dectin-1	0.760 (0.709–0.811)	0.047 (0.023–0.072)	0.010^†^	0.763 (0.713–0.814)	0.040 (0.001–0.080)	0.050^†^
Nomogram	0.796 (0.747–0.846)	—		0.812 (0.769–0.856)	—	
Nomogram vs SSIGN						
in all patients	—	0.086 (0.435–0.129)	<0.001^‡^	—	0.072 (0.036–0.110)	<0.001^‡^
in SSIGN low/intermediate groups	—	0.097 (0.049–0.146)	<0.001^‡^	—	0.099 (0.053–0.146)	<0.001^‡^
Nomogram vs UISS						
in all patients	—	0.082 (0.045–0.119)	<0.001^‡^	—	0.089 (0.043–0.135)	<0.001^‡^
in UISS low/intermediate groups	—	0.095 (0.069–0.130)	<0.001^‡^	—	0.092 (0.052–0.133)	<0.001^‡^

^†^Compared the c-index with the original model without tumoral Dectin-1 expression data; ^‡^Compared the c-index of nomogram with SSIGN/UISS stratification in different patient groups; C-index = concordance index; CI = confidence interval; SSIGN = Mayo clinic stage, size, grade, and necrosis score; UISS = UCLA Integrated Staging System. C-index and 95%CI were calculated from 1000 bootstrap samples to protect from overfitting.
