# Supplementary material for: Dectin-1 predicts adverse postoperative prognosis of patients with clear cell renal cell carcinoma
Source: Sci Rep. 2016 Sep 7;6:32657. doi: 10.1038/srep32657 (PMC5013447; doi:10.1038/srep32657)
Supplement: Supplementary Information [file srep32657-s1.doc]

**Dectin-1 predicts adverse postoperative prognosis of patients with clear cell renal cell carcinoma**

Yu Xia^1,3^, Li Liu^1,3^, Qi Bai^1,3^, Jiajun Wang^1^, Wei Xi^1^, Yang Qu^1^, Ying Xiong^1^, Qilai Long^1^, Jiejie Xu^2,*^, Jianming Guo^1,*^

^1^Department of Urology, Zhongshan Hospital, Fudan University, Shanghai 200032, China;

^2^Department of Biochemistry and Molecular Biology, School of Basic Medical Sciences, Fudan University, Shanghai 200032, China;

^3^These authors contributed equally to this work.

***Corresponding authors.** Department of Biochemistry and Molecular Biology, School of Basic Medical Sciences, Fudan University, Shanghai 200032, China. E-mail address: jjxufdu@fudan.edu.cn (**J. Xu**) or Department of Urology, Zhongshan Hospital, Fudan University, Shanghai 200032, China. E-mail address: guo.jianming@zs-hospital.sh.cn (**J. Guo**)

| **Table S1: Univariate analyses of characteristics associated with recurrence free survival and overall survival** | | | | | | | |
| --- | --- | --- | --- | --- | --- | --- | --- |
| **Variables** | **RFS (n=265)** | | |  | **OS (n=290)** | | |
|  | **Hazard Ratio** | **95%CI** | **P-value**† |  | **Hazard Ratio** | **95%CI** | **P-value**† |
| Age, years* |  |  |  |  |  |  |  |
| >55 *vs* ≤55 | 1.777 | 1.109-2.846 | 0.017 |  | 2.059 | 1.311-3.234 | 0.002 |
| Gender |  |  |  |  |  |  |  |
| Male *vs* Female | 1.009 | 0.615-1.657 | 0.970 |  | 1.081 | 0.677-1.729 | 0.744 |
| Tumor size* |  |  |  |  |  |  |  |
| >4cm *vs* ≤4cm | 2.251 | 1.409-3.598 | 0.001 |  | 2.038 | 1.315-3.157 | 0.001 |
| Pathological T stage |  |  | <0.001 |  |  |  | <0.001 |
| pT1 | 1.000 | reference | - |  | 1.000 | reference | - |
| pT2 | 3.469 | 1.698-7.088 | 0.001 |  | 3.503 | 1.837-6.681 | <0.001 |
| pT3 | 2.938 | 1.752-4.926 | <0.001 |  | 3.371 | 2.084-5.452 | <0.001 |
| pT4 | 14.899 | 5.151-43.096 | <0.001 |  | 7.870 | 2.397-25.845 | 0.001 |
| Pathological N stage |  |  |  |  |  |  |  |
| pN1 *vs* pN0 | - | - | - |  | 1.145 | 0.150-8.728 | 0.896 |
| Distant metastasis |  |  |  |  |  |  |  |
| Yes *vs* No | - | - | - |  | 5.324 | 2.882-9.833 | <0.001 |
| TNM stage |  |  | <0.001 |  |  |  | <0.001 |
| I | 1.000 | reference | - |  | 1.000 | reference | - |
| II | 3.709 | 1.860-7.394 | <0.001 |  | 3.242 | 1.574-6.681 | 0.001 |
| III | 2.865 | 1.694-4.813 | <0.001 |  | 3.437 | 2.051-5.759 | <0.001 |
| IV | 14.914 | 5.156-43.143 | <0.001 |  | 9.324 | 4.967-17.501 | <0.001 |
| Fuhrman grade |  |  | <0.001 |  |  |  | <0.001 |
| 1-2 | 1.000 | reference | - |  | 1.000 | reference | - |
| 3 | 3.355 | 1.992-5.652 | <0.001 |  | 3.143 | 1.944-5.082 | <0.001 |
| 4 | 4.775 | 1.486-15.344 | 0.009 |  | 4.308 | 1.347-13.770 | 0.014 |
| Necrosis |  |  |  |  |  |  |  |
| Present *vs* Absent | 3.255 | 1.940-5.462 | <0.001 |  | 2.885 | 1.769-4.705 | <0.001 |
| ECOG PS |  |  | <0.001 |  |  |  | <0.001 |
| 0 | 1.000 | reference | - |  | 1.000 | reference | - |
| 1 | 2.681 | 1.614-4.455 | <0.001 |  | 3.128 | 1.980-4.941 | <0.001 |
| 2 | 3.107 | 1.111-8.692 | 0.031 |  | 2.822 | 1.116-7.137 | 0.028 |
| 3 | 7.942 | 2.815-22.405 | <0.001 |  | 5.068 | 1.566-16.404 | 0.007 |
| Tumoral dectin-1 |  |  |  |  |  |  |  |
| High *vs* Low | 3.139 | 1.953-5.045 | <0.001 |  | 3.053 | 1.966-4.742 | <0.001 |
| *Split at median; ECOG PS= Eastern Cooperative Oncology Group performance status; CI=confidence interval; OS= overall survival; RFS= recurrence free survival; †Data obtained from the Cox proportional hazards model, P**-**value <0.05 was regarded as statistically significant | | | | | | | |
